# First and subsequent asbestos exposures in relation to mesothelioma and lung cancer mortality

**DOI:** 10.1038/sj.bjc.6603998

**Published:** 2007-09-25

**Authors:** E Pira, C Pelucchi, P G Piolatto, E Negri, G Discalzi, C La Vecchia

**Affiliations:** 1 Dipartimento di Traumatologia, Ortopedia e Medicina del Lavoro, Università degli Studi di Torino, via Zuretti 29, Torino 10126, Italy; 2 Istituto di Ricerche Farmacologiche ‘Mario Negri’, via Eritrea 62, Milano 20157, Italy; 3 Istituto di Statistica Medica e Biometria ‘GA Maccacaro’, Università degli Studi di Milano, via Venezian 1, Milano 20133, Italy

**Keywords:** asbestos, cancer mortality, cohort studies, lung cancer, mesothelioma, occupational exposure

## Abstract

We analysed data from a cohort of 1966 subjects (889 men and 1077 women) employed by an Italian asbestos (mainly textile) company in the period 1946–1984, who were followed-up to 2004. A total of 62 025 person-years of observation were recorded. We computed standardised mortality ratios (SMR) for all causes and selected cancer sites using national death rates for each 5-year calendar period and age group. There were 68 deaths from mesothelioma (25 men and 43 women, 39 pleural and 29 peritoneal) *vs* 1.6 expected (SMR=4159), and 109 from lung cancer *vs* 35.1 expected (SMR=310). The SMRs of pleural/peritoneal cancer were 6661 for subjects exposed only before 30 years of age, 8019 for those first exposed before 30 and still employed at 30–39 years of age and 5786 for those first exposed before 30 and still employed at 40 or more years of age. The corresponding SMRs for lung cancer were 227, 446 and 562. The SMR of mesothelioma was strongly related to time since first exposure. The SMR of lung cancer, but not of mesothelioma, appeared to be related to subsequent exposures.

The incidence of mesothelioma rises as function of the third or fourth power of time since first asbestos exposure (latency) (Peto *et al*, 1982). Models of asbestos-related mesothelioma carcinogenesis also include a direct relation with average exposure to asbestos fibres, and a term for type of asbestos, with risk apparently higher for amphiboles than chrysotile (Boffetta, 1998). The influence of other time-related aspects such as age at first exposure and duration of exposure is largely or totally explained by latency (Peto *et al*, 1982; Pira *et al*, 2005), but the role of early and later occupational exposures to asbestos requires further investigation. Recently, fewer deaths from mesothelioma have been reported than predicted on the basis of models of carcinogenesis (Berry *et al*, 2004; Pelucchi *et al*, 2004), raising the possibility of a gradual elimination of asbestos from the lungs (Berry *et al*, 2004). Asbestos is also an established cause of lung cancer (IARC, 1987) with risk related to duration of exposure and cumulative dose.

We previously examined follow-up data to the end of 1996 from a cohort of nearly 2000 subjects (including more than 1000 women) formerly employed in an Italian asbestos factory (Pira *et al*, 2005). We have now extended the follow-up to the end of 2004, with 9000 additional person-years of observation allowing us to consider the role of latency, and attempt separation of first from subsequent exposures, in relation to mesothelioma and lung cancer risks.

## MATERIALS AND METHODS

Included in the cohort were 1966 subjects (889 males and 1077 females) who had worked in a major asbestos textile factory for at least 1 month in the period from 1946 to 1984, when production ceased (Pira *et al*, 2005). Various types of asbestos were used in the factory, including crocidolite. For the present analyses, follow-up was stopped on 31 December 2004 or at turning 80 years of age, if this occurred earlier. Overall, 1132 (57.6%) subjects survived to the end of the study period, 730 (37.1%) died and 104 (5.3%) emigrated or were lost to follow-up, these being included up to the latest date of information. A total of 62 025 person-years of observation (25 139 man-years and 36 886 woman-years) were included in the present analysis.

We obtained employment data from personnel records at the factory, and ascertained vital status and causes of death through registers in municipal offices and local health units. Information was available on date and place of birth, migration(s) and death, on cause of death and on periods and duties of employment(s). We further checked subjects against regional mesothelioma registers.

We computed expected numbers of deaths from all causes and from selected cancer sites using national death rates for each 5-year calendar period and age group. The latter were available only since 1955 from the Italian National Institute of Statistics (ISTAT, 1955–2002) and the World Health Organization (WHO, 2006), so 1955–1959 death rates were also applied for 1946–1949 and 1950–1954. As Italian death rates were not yet available for years 2003–2004, we applied those for 2002 (the mid-year) to the whole period 2000–2004. In 1995, ISTAT introduced automatic coding of causes of death, and though for most causes this introduced no material effect, for a few cancer sites – including peritoneum – mortality was not comparable with earlier calendar periods. For peritoneal cancer, therefore, we used the 1990–1994 rates for 1995–1999 and 2000–2004. We computed the standardised mortality ratios (SMR) of selected cancers for the overall study population, separately for men and women (Breslow and Day, 1987).

## RESULTS

Table 1 gives observed and expected numbers of deaths, and the corresponding SMRs and 95% confidence intervals (CI) for major cancer sites, separately for men and women. Overall, there were 730 deaths compared with 424.5 expected (SMR=172), and 325 cancer deaths compared with 153.9 expected (SMR=211). The SMRs for all neoplasms were 194 for males and 244 for females, and the corresponding SMRs for all causes of death were 162 and 194. Sixty-eight subjects (25 men and 43 women) died from pleural or peritoneal cancer, as compared to 1.6 expected. Corresponding SMRs were 4623 for pleural and 3665 for peritoneal cancers. Significant excess risks were reported also for lung (SMR=310), liver (SMR=237), laryngeal (SMR=236) and ovarian (SMR=283) cancers. For pleural, peritoneal and lung cancer, the SMRs were higher in women than in men.

Table 2 shows the number of observed and expected deaths from pleural/peritoneal and lung cancers, and the corresponding SMRs, according to time-related factors. For an early age at first employment, the SMR of pleural/peritoneal cancers was 7968, whereas when first employment occurred at 35 years of age or more, the SMR was 2085. No trend in risk of lung cancer was observed with age at first employment, and the highest SMR was 424 for subjects in the intermediate category (ie first employment between 25 and 34 years of age). With reference to duration of employment, an increasing trend was evident for lung cancer, but not for mesothelioma. For both neoplasms, the SMRs were higher after 15 years since first employment. No trend in risk was found with time since last employment. The risk of mesothelioma was highest in subjects first employed between 1965 and 1974 (SMR=7331).

Table 3 shows the number of observed and expected deaths from pleural/peritoneal cancer and the corresponding SMRs according to time-related factors, in strata of period at first employment. In both strata, mesothelioma was inversely associated with age at first employment in the factory, and directly with latency (SMRs=3372 and 10 903 for ⩾30 years since first employment in subjects first employed before 1965 and in 1965–1974, respectively). With reference to duration of exposure, there was a linear trend in risk for subjects first employed before 1965, but not for those first employed during 1965–1974. No clear relation emerged with time since last employment in both strata. SMRs were higher for subjects with first employment between 1965 and 1974 than for those first employed before 1965.

Table 4 gives observed and expected numbers of deaths from pleural/peritoneal and lung cancers, and their SMRs, according to age at first and subsequent employment, overall and in strata of sex. The SMRs of pleural/peritoneal cancers were 6661 for subjects employed only before 30 years of age, 8019 for those first exposed before 30 and still employed at 30–39 years of age and 5786 for those first exposed before 30 and still employed at 40 or more years of age. Risk was lower with first employment at age 30–39 (SMR=3559) and ⩾40 years of age (SMR=1943). For lung cancer, the SMRs increased from 227 for subjects employed only before 30 years to 446 for subjects first exposed before 30 and still employed at 30–39 years of age and to 562 for those first exposed before 30 and still employed at 40 or more years of age. The SMRs were 388 and 243 for first employment at 30–39 and ⩾40 years of age, respectively. All SMRs were higher in women than in men, due to their lower baseline risks, but the patterns of risk with time at first and last employment were similar.

Table 5 gives observed and expected mortality from pleural/peritoneal cancer, combining employment before and after age 30 years. Among subjects with no employment before age 30, the SMRs tended to increase with longer duration of employment after age 30 (SMR=4564 for ⩾10 years of employment). On the other hand, among those first employed before age 30, no such trend in risk emerged for duration of exposure after age 30, as SMRs were 8075 and 5429 for durations of <10 and ⩾10 years, respectively.

## DISCUSSION

As compared to the 1996 follow-up (Pira *et al*, 2005), in this updated analysis there were 31 additional deaths from pleural and peritoneal mesothelioma (total 68), and 29 additional deaths from lung cancer (total 109), allowing examination of the role of first and subsequent asbestos exposures in this factory on mesothelioma and lung cancer risk. The SMR of pleural/peritoneal mesothelioma continued to use in the years 1996–2004, the total number of such deaths having almost doubled. Thus, the overall SMR for all mesotheliomas (pleural and peritoneal) rose from 3139 in 1946–1996 to 6792 in 1997–2004. This points to longer latency as the key factor in mesothelioma risk, consistent with models that describe incidence as a power function of time since first asbestos exposure (Peto *et al*, 1982). For lung cancer, the SMR only changed modestly from 282 to 310 over the last 8 years considered. The appreciable fall in asbestos exposures since the early 1970s in the factory (Pira *et al*, 2005) and the cessation of exposure in 1984 may have contributed to this levelling of lung cancer risk, and further supports the view that time-related factors involved in mesotheliomas and lung cancers differ.

When we stratified our data according to the period at first employment, direct trends emerged with latency. In 1968 the acquisition of a nearby asbestos manufacture factory with movement of some of workers between plants and hence a lack of information on previous exposures of these subjects might explain the inconsistent results. Although an occupational history was not taken, any available data on other asbestos-related employments were recorded. The elevated risks of mesothelioma found with latency <15 years (SMR=685) and age at first employment ⩾40 years (SMR=1943) are likely to be due to previous asbestos exposures in other occupational settings. In fact, both subjects who died from pleural mesothelioma before 15 years after first employment had previous job-related exposures. Past asbestos exposure was also recorded for another worker reporting first employment in the plant after 40 years of age. Thus, mesothelioma risk for a late age at first exposure was overestimated.

The excess of laryngeal cancer in our data is in agreement with a report from the US Institute of Medicine (IOM, 2006), which concluded that there is sufficient evidence for an association between asbestos exposure and laryngeal cancer. This was based both on the consistency of findings of epidemiological studies and the biological plausibility of such an association.

Some studies of occupational asbestos exposures reported increases in risk of digestive, kidney and ovarian cancers, but results have been inconsistent (Doll and Peto, 1985; Seidman *et al*, 1986; IARC, 1987; Smith *et al*, 1989; Berry *et al*, 2000; Reid *et al*, 2004), and the evidence was later considered suggestive but not sufficient (IOM, 2006). We found increased mortality from ovarian and liver cancers. Misdiagnosis of peritoneal cancers (La Vecchia, 2001; Boffetta, 2007) may explain the excess of ovarian (as well as the small excess of intestinal) cancer deaths, and misdiagnosis of secondaries that of liver cancer. Our findings weigh against an association between asbestos and kidney cancer.

Using mortality data from the province of Turin, when available (ie for years 1980–2000) (ASL 5, 2004), we found the SMRs of pleural cancer were lower, particularly in women, than those using national reference rates, but were still extremely high (2039 in men, 6431 in women and 3414 overall). Corresponding data for peritoneal cancer were not available. For lung as well as for many other cancer sites of interest, rates in Turin do not appreciably differ from Italian ones, and thus risk estimates were almost unchanged (SMRs of lung cancer were 268 in men, 629 in women and 313 overall). The higher mesothelioma SMRs in women than men are likely to be attributable to the lower baseline rates in women.

Among possible sources of bias, is the validity of SMRs, as a risk indicator (Rothman and Greenland, 1998; Goldman and Brender, 2000). Direct standardisation is preferable in general (MacMahon and Trichopoulos, 1996), but inappropriate here because of the relatively small number of mesothelioma deaths (68). Cohort studies may also underestimate the true risk when SMRs are large (Jones and Swerdlow, 1998). Strengths of our study are the long period of observation, the small number of individuals lost to follow-up, and the inclusion of many women (Boffetta, 2007). Further, many subjects (particularly women) had been exposed to large amounts of asbestos dusts for short time periods (Pira *et al*, 2005), thereby allowing separation of the roles of early from that of later exposure.

The main finding of this study was that, for pleural and peritoneal cancers, the SMR did not increase in subjects reporting both earlier and later asbestos exposures. In fact, they were similar for those employed only before 30 years and for those employed both before 30 and after 39 years, whereas the lower risks in subjects with an older age at first employment are attributable to shorter latency. In contrast, for lung cancer subsequent exposures led to appreciable increments in relative risks.

## Figures and Tables

**Table 1:** Observed and expected deaths from major cancers and corresponding standardised mortality ratios (SMR) and 95% confidence intervals (CI)

	**Males**			**Females**			**Total**			
**Cause of death**	**Observed**	**Expected**	**SMR**	**Observed**	**Expected**	**SMR**	**Observed**	**Expected**	**SMR**	**95% CI**
Oral and pharyngeal cancer	7	3.3	215	0	0.5	0	7	3.8	184	74–380
Esophageal cancer	2	2.4	85	2	0.3	603	4	2.7	149	41–381
Stomach cancer	12	10.8	111	5	3.9	128	17	14.7	115	67–185
Colorectal cancer	15	9.4	159	8	5.9	135	23	15.4	150	95–224
Liver cancer	8	3.5	226	3	1.1	274	11	4.6	237^a^	118–425
Peritoneal cancer	9	0.4	2113^a^	20	0.4	5473^a^	29	0.8	3665^a^	2455–5277
Laryngeal cancer	8	3.3	245^a^	0	0.1	0	8	3.4	236^a^	102–464
Lung cancer	82	31.0	265^a^	27	4.2	647^a^	109	35.1	310^a^	255–374
Pleural cancer	16	0.6	2626^a^	23	0.2	9809^a^	39	0.8	4623^a^	3287–6319
Breast cancer	—	—	—	9	12.1	75	9	12.1	75	34–142
Ovarian cancer	—	—	—	8	2.8	283^a^	8	2.8	283^a^	122–557
Prostate cancer	4	4.4	90	—	—	—	4	4.4	91	25–232
Bladder cancer	5	4.0	125	0	0.5	0	5	4.5	112	36–260
Kidney cancer	0	2.0	0	3	0.7	408	3	2.8	108	22–316
Lymphatic/hematopoietic cancer	3	6.5	46	5	4.3	115	8	10.9	74	32–145
Pleural+peritoneal cancer	25	1.0	2415^a^	43	0.6	7167^a^	68	1.6	4159^a^	3229–5273
All cancers	195	100.7	194^a^	130	53.3	244^a^	325	153.9	211^a^	189–235
All causes	476	293.3	162^a^	254	131.1	194^a^	730	424.5	172^a^	160–185
Total no. of subjects	889			1077			1966			
Person-years	25 139			36 886			62 025			

a 95% CI does not include 100.

**Table 2:** Observed and expected deaths from pleural/peritoneal and lung cancers and corresponding standardised mortality ratios (SMR), according to time-related characteristics of employment

		**Pleural/peritoneal cancers**			**Lung cancer**		
**Covariate**	**Person-years**	**Observed**	**Expected**	**SMR**	**Observed**	**Expected**	**SMR**
*Age at first employment (years)*							
<25	29 570	29	0.4	7968^a^	14	5.0	281^a^
25–34	17 048	22	0.5	4828^a^	38	9.0	424^a^
⩾35	15 408	17	0.8	2085^a^	57	21.2	269^a^
							
*Duration of employment (years)*							
<1	22 473	10	0.5	1897^a^	20	12.2	164^a^
1–<5	18 741	23	0.4	5469^a^	25	8.7	288^a^
5–<10	10 682	12	0.3	4127^a^	16	5.9	270^a^
⩾10	10 129	23	0.4	5801^a^	48	8.4	571^a^
							
*Time since first employment (years)*							
<15	25 282	2^b^	0.3	685	6	6.7	90
15–<30	23 887	33	0.7	4913^a^	58	15.5	375^a^
⩾30	12 856	33	0.7	4915^a^	45	13.0	346^a^
							
*Time since last employment (years)*							
<15	35 201	12	0.6	1991^a^	42	13.9	302^a^
15–<25	13 948	30	0.4	6842^a^	34	9.4	363^a^
⩾25	12 877	26	0.6	4378^a^	33	11.9	278^a^
							
*Period at first employment*							
Before 1955	13 368	11	0.4	2744^a^	17	7.6	225^a^
1955–1964	20 513	11	0.6	1931^a^	40	12.9	309^a^
1965–1974	26 501	46	0.6	7331^a^	52	13.7	379^a^
1975 or later	1644	0	0.04	0	0	0.9	0

a 95% confidence interval does not include 100.

b Occupational exposure to asbestos before employment in the plant was recorded for both subjects.

**Table 3:** Observed and expected deaths from pleural/peritoneal cancer and corresponding standardised mortality ratios (SMR), according to time-related characteristics of employment in strata of period at first employment

	**First employment before 1965**				**First employment between 1965 and 1974**			
**Covariate**	**Person-years**	**Observed**	**Expected**	**SMR**	**Person-years**	**Observed**	**Expected**	**SMR**
*Age at first employment (years)*								
<25	17 166	13	0.28	4688^a^	11 599	16	0.08	19 134^a^
25–34	9170	8	0.30	2644^a^	7529	14	0.15	9409^a^
⩾35	7545	1	0.39	256	7373	16	0.40	4050^a^
								
*Duration of employment (years)*								
<1	11 094	3	0.30	988^a^	10 956	7	0.22	3189^a^
1–<5	9798	6	0.23	2583^a^	8141	17	0.17	9833^a^
5–<10	6126	4	0.16	2527^a^	4136	8	0.11	6969^a^
⩾10	6862	9	0.28	3258^a^	3267	14	0.12	11 643^a^
								
*Time since first employment (years)*								
<15	11 913	0	0.11	0	12 431	2	0.17	1197^a^
15–<30	12 141	4	0.33	1229^a^	11 040	29	0.32	8983^a^
⩾30	9827	18	0.53	3372^a^	3029	15	0.14	10 903^a^
								
*Time since last employment (years)*								
<15	18 205	1	0.31	326	15 848	11	0.28	3998^a^
15–<25	6942	10	0.23	4350^a^	6544	20	0.19	10 358^a^
⩾25	8734	11	0.43	2536^a^	4110	15	0.16	9421^a^
All subjects	33 881	22	0.97	2267^a^	26 501	46	0.63	7331^a^

a 95% confidence interval does not include 100.

**Table 4:** Observed and expected deaths from pleural/peritoneal and lung cancers and corresponding standardised mortality ratios (SMR), according to age at first employment and subsequent history of employment

		**Pleural/peritoneal cancers**			**Lung cancer**		
	**Person-years**	**Observed**	**Expected**	**SMR**	**Observed**	**Expected**	**SMR**
*Males*							
First and last employment <30 years of age	8367	4	0.17	2303^a^	7	4.2	166
First employment <30 years of age, last employment 30–39 years of age	1992	3	0.05	5827^a^	3	1.4	221
First employment <30 years of age, last employment ⩾40 years of age	1086	2	0.05	3730^a^	6	1.7	363^a^
First employment 30–39 years of age	6807	7	0.29	2392^a^	30	8.8	341^a^
First employment ⩾40 years of age	6887	9^b^	0.46	1941^a^	36	15.0	241^a^
							
*Females*							
First and last employment <30 years of age	21 972	21	0.20	10 413^a^	6	1.5	392^a^
First employment <30 years of age, last employment 30–39 years of age	4043	6	0.06	9877^a^	5	0.4	1154^a^
First employment <30 years of age, last employment ⩾40 years of age	1774	4	0.05	7987^a^	5	0.3	1639^a^
First employment 30–39 years of age	6726	10	0.18	5406^a^	9	1.2	720^a^
First employment ⩾40 years of age	2372	2	0.10	1952^a^	2	0.7	306
							
*All subjects*							
First and last employment <30 years of age	30 338	25	0.38	6661^a^	13	5.7	227^a^
First employment <30 years of age, last employment 30–39 years of age	6035	9	0.11	8019^a^	8	1.8	446^a^
First employment <30 years of age, last employment ⩾40 years of age	2860	6	0.10	5786^a^	11	2.0	562^a^
First employment 30–39 years of age	13 533	17	0.48	3559^a^	39	10.0	388^a^
First employment ⩾40 years of age	9259	11^b^	0.57	1943^a^	38	15.6	243^a^

a 95% confidence interval does not include 100.

b Occupational exposure to asbestos before employment in the plant was recorded for three subjects.

**Table 5:** Observed and expected deaths from pleural/peritoneal cancer and corresponding standardised mortality ratios (SMR), according to employment before age 30 and duration of employment at age ⩾30 years

	**Duration of employment at age ⩾30 years**								
	**None**			**<10 years**			**⩾10 years**		
	**Person-years**	**O : E**	**SMR**	**Person-years**	**O : E**	**SMR**	**Person-years**	**O : E**	**SMR**
*Employment before age 30*									
No	—	—	—	17 999	16 : 0.78	2049^a^	4793	12 : 0.26	4564^a^
Yes	30 338	25 : 0.38	6661^a^	6376	10 : 0.12	8075^a^	2519	5 : 0.09	5429^a^

a 95% confidence interval does not include 100.
